# Aeromedical decision-making for asymptomatic cerebral cavernous malformations in military pilots

**DOI:** 10.3389/fpubh.2026.1794579

**Published:** 2026-04-16

**Authors:** Hui Zhang, Li Ma, Cheng-Ye Zhang, Jian Zhou, Yan Zhou, Yu-Dong Ma

**Affiliations:** 1 Department of Neurosurgery, Air Force Medical Center, Beijing, China; 2 Department of Anesthesiology, 7th Medical Center of the Chinese PLA General Hospital, Beijing, China

**Keywords:** aeromedical decision-making, asymptomatic state, cerebral cavernous malformation, military pilot, risk stratification

## Abstract

**Introduction:**

The increasing incidental discovery of asymptomatic cerebral cavernous malformations (CCMs) in military pilots poses a significant challenge for aeromedical certification, due to the potential risks of epilepsy and symptomatic hemorrhage. There is a lack of evidence-based guidelines for the fitness-to-fly assessment of military pilots with such lesions.

**Methods:**

A retrospective study was conducted on military pilots diagnosed with asymptomatic CCMs at our center. Demographic, flight-related, and lesion data were collected. A multi-disciplinary review board evaluated in-flight incapacitation risk by assessing epilepsy and hemorrhage potential based on established clinical risk factors.

**Results:**

Among 22 military pilots harboring 23 CCMs, 11 were permanently disqualified from flight duties due to a high assessed risk of in-flight incapacitation. This included five pilots disqualified for cortical lesions posing a significant epilepsy risk, and six pilots disqualified due to a high risk of symptomatic hemorrhage associated with brainstem location, eloquent area involvement, or Zabramski Type II lesions. The remaining 11 pilots, with solitary Zabramski Type III or IV lesions that were neither cortical nor located in eloquent areas, were approved for unrestricted flying status. During a mean follow-up of 34 months, these pilots demonstrated stable lesion morphology on imaging and experienced no neurological events.

**Conclusion:**

A risk-stratified aeromedical certification protocol for asymptomatic CCMs in military pilots appears viable. Strict disqualification for high-risk lesions combined with conditional clearance for low-risk lesions, supported by rigorous annual monitoring, can balance flight safety with career preservation. This preliminary framework requires further validation.

## 1 Introduction

Cerebral cavernous malformations (CCMs) are vascular anomalies with a 0.5% population prevalence (1, 2). CCMs are the most frequent incidental cerebrovascular abnormalities in Chinese military aircrew, followed by intracranial aneurysms (3). While many CCMs remain asymptomatic, they carry a risk of symptomatic hemorrhage or seizures, either of which can lead to neurological deficits (2). For aviation personnel, even a minor neurological event could precipitate catastrophic consequences in the flight enviroment, directly jeopardizing aviation safety. Consequently, the aeromedical disposition of aviators with CCMs necessitates a rigorous, evidence-based approach to fitness-to-fly evaluation. This process must carefully balance two competing imperatives: excessive conservatism may result in the unnecessary grounding of otherwise fit and proficient aviators, incurring significant personal and operational costs; conversely, an inadequate assessment of risk could fail to identify genuinely hazardous conditions, thereby compromising flight safety.

This study presents a retrospective analysis of asymptomatic CCMs in military pilots. By evaluating hemorrhage rates, seizure incidence, and key lesion characteristics, we aim to establish evidence-based fitness-to-fly criteria. The ultimate goal is to provide data-driven guidance that effectively balances operational flight safety with the preservation of aviation careers.

## 2 Methods

### 2.1 Study design

All military pilots diagnosed with CCMs at our center were included in this retrospective study. Descriptive statistics were used to summarize the cohort characteristics. Collected variables included demographic data (age), flight-related information (aircraft type), CCM lesion characteristics (size and anatomic location), Zabramski classification (4), clinical follow-up duration, and final aeromedical disposition. Furthermore, the structured aeromedical evaluation process—a multi-stage assessment conducted by a dedicated review board and involving specialists in neurology, neurosurgery, radiology, and aerospace medicine—is described in detail in the following section. This study was approved by the local institutional review board and the local ethics commission (No. KT2025-65-PJ01).

### 2.2 Inclusion and exclusion criteria

The inclusion criteria for participants were as follows: (1) patients hospitalized in our center from November 2020 to October 2025; (2) active military pilots; (3) availability of complete imaging data; (4) pilots meeting the diagnostic criteria for asymptomatic CCM. Subjects were required to meet all of the above criteria simultaneously.

The exclusion criteria for participants were as follows: (1) ground crews (including air traffic controllers, photographers, flight attendants, etc.); (2) Patients who refuse to fly or fail to complete aviation medical evaluation. Patients with any of the above conditions were excluded.

### 2.3 Aeromedical evaluation

Irrespective of the treatment received, flight readiness must be assessed by the specialists at a dedicated aeromedical review board. First, a multidisciplinary team comprising neurologist, neurosurgeons and radiology specialists determine appropriate intervention (surgical or conservative management). Subsequently, for CCMs deemed suitable for conservative management, a second evaluation is conducted by specialists in neurology, neurosurgery, and aerospace medicine to assess the in-flight incapacitation risk. For CCMs, the focus is on analyzing their risks of CCM-related epilepsy and symptomatic hemorrhage, with further assessment of the risk of in-flight incapacitation based on the pilot's aircraft type and role during missions (5). Final aeromedical disposition is then determined based on this specific risk.

## 3 Results

### 3.1 Clinical features

**Figure 1:**
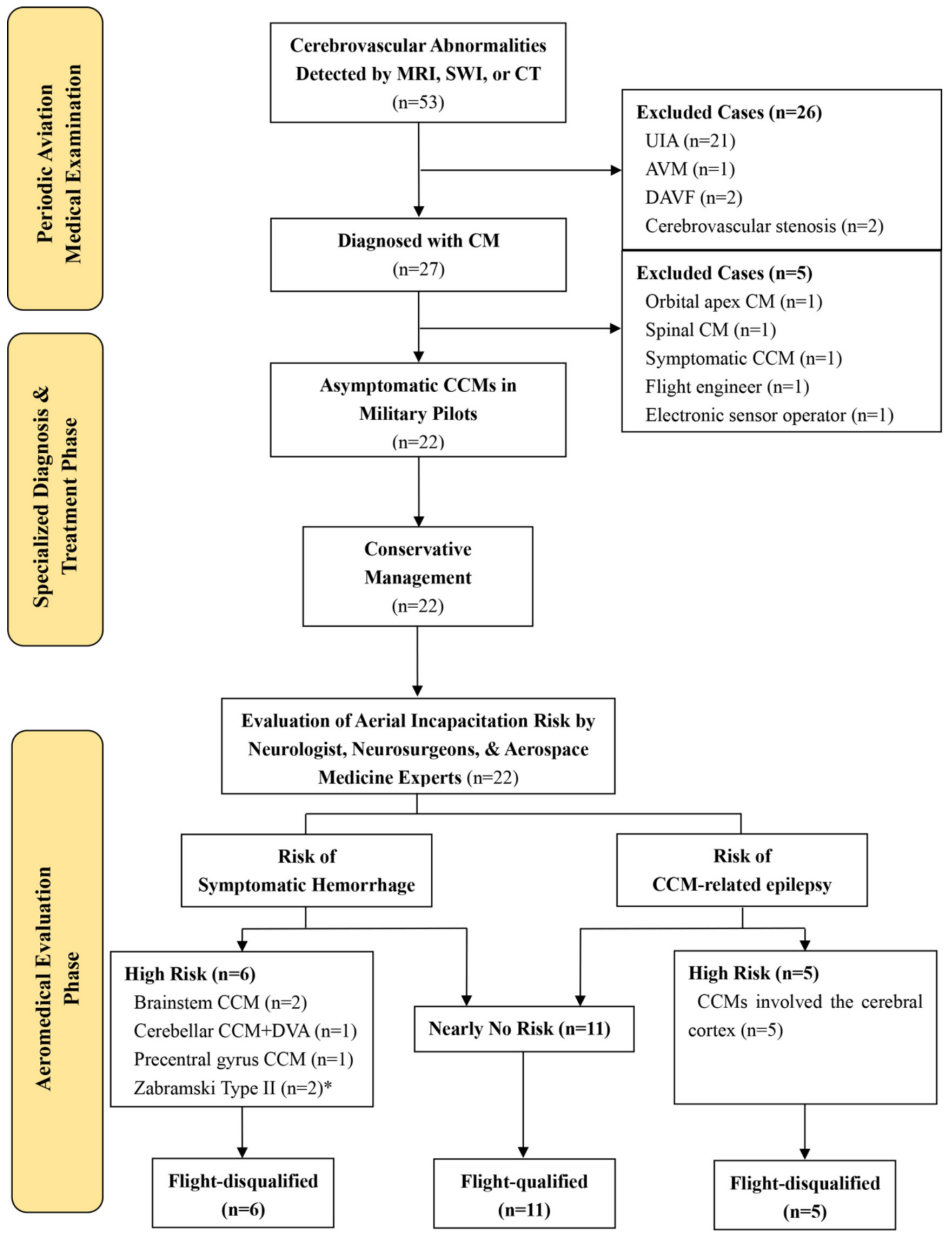
Flow diagram of fitness-to-fly assessment in asymptomatic CCMs. *Zabramski Type II CCMs located within the white matter of a cerebral lobe.

A total of 53 aircrew members were found to have cerebrovascular anomalies during their annual aviation medical examination, which included routine brain MRI, susceptibility-weighted imaging (SWI), and head CT. After excluding 21 cases of unruptured intracranial aneurysms, one case of brain arteriovenous malformation, two cases of dural arteriovenous fistula, and two cases of cerebrovascular stenosis, 27 cases of cavernous malformations were identified. Further exclusion of one case of orbital apex cavernous malformation (case 25), one case of spinal cavernous malformation (case 22), one case of CCM presenting with acute hemorrhage (case 24), one flight engineer, and one airborne electronic sensor operator resulted in a final cohort of 22 military pilots with asymptomatic CCMs (see Figure 1 and Table 1).

**Table 1:** Key characteristics of the military pilots with CCMs.

Case	Age (year)	Sex	Somker	Hypertension	Aircraft types	Symptoms & Signs	Anatomical location	Eloquent area	Size	Forms of CCM	Lesion multiplicity	Presence of DVA	Zabramskiclassification	EEG	Treatment	Risk of symptomatic hemorrhage	Risk of epilepsy	Aviation medicalcertification	Follow-up after aviation medical certification
1	45	M	Never	No	Jet fighter	Absent	Left parietal lobe (subcortex)	No	1 mm	Sporadic	Single lesion	No	III	Normal	Conservative management	Nearly no risk	Nearly no risk	Flight-qualified	12 m
2	35	M	Never	No	Jet fighter	Absent	Right frontal lobe (subcortex)	No	4 mm	Sporadic	Single lesion	No	III	Normal	Conservative management	Nearly no risk	Nearly no risk	Flight-qualified	32 m
3	33	M	Never	No	Helicopter	Absent	Right frontal lobe (subcortex)	No	4 mm	Sporadic	Single lesion	No	III	Normal	Conservative management	Nearly no risk	Nearly no risk	Flight-qualified	39 m
4	43	M	Never	No	Jet fighter	Absent	Left parietal lobe (subcortex)	No	-	Sporadic	Single lesion	No	IV	Normal	Conservative management	Nearly no risk	Nearly no risk	Flight-qualified	48 m
5	28	M	Never	No	Jet fighter	Absent	Left frontal lobe (subcortex)	No	-	Sporadic	Single lesion	No	IV	Normal	Conservative management	Nearly no risk	Nearly no risk	Flight-qualified	31 m
6	36	M	Never	No	Jet fighter	Absent	Right temporal pole (subcortex)	No	2.5 mm	Sporadic	Single lesion	No	III	Normal	Conservative management	Nearly no risk	Nearly no risk	Flight-qualified	40 m
7	35	M	Never	No	Jet fighter	Absent	Right frontal lobe (subcortex)	No	-	Sporadic	Single lesion	No	IV	Normal	Conservative management	Nearly no risk	Nearly no risk	Flight-qualified	38m
8	36	M	Never	No	Jet fighter	Absent	Right frontal lobe (subcortex)	No	-	Sporadic	Single lesion	No	IV	Normal	Conservative management	Nearly no risk	Nearly no risk	Flight-qualified	25 m
9	30	M	Never	No	Jet fighter	Absent	Right frontal lobe (subcortex)	No	3.5	Sporadic	Single lesion	No	III	Normal	Conservative management	Nearly no risk	Nearly no risk	Flight-qualified	53 m
10	21	M	Never	No	Jet fighter	Absent	Right frontal lobe (subcortex)	No	–	Sporadic	Single lesion	No	IV	Normal	Conservative management	Nearly no risk	Nearly no risk	Flight-qualified	40 m
11	31	M	Never	No	Jet fighter	Absent	Right temporal lobe (subcortex)	No	–	Sporadic	Single lesion	No	IV	Normal	Conservative management	Nearly no risk	Nearly no risk	Flight-qualified	13 m
12	21	M	Never	No	Jet Fighter	Absent	Right temporal lobe (subcortex)	No	8 mm	Sporadic	Single lesion	No	II	Normal	Conservative management	High risk	–	Flight-disqualified	–
13	37	M	Never	No	Jet fighter	Absent	Right frontal lobe (subcortex)	No	5 mm	Sporadic	Single lesion	No	II	Normal	Conservative management	High risk	–	Flight-disqualified	–
14	43	M	Never	No	Jet fighter	Absent	Right frontal lobe (subcortex)	Precentral gyrus	12 mm	Sporadic	Single lesion	No	II	Normal	Conservative management	High risk	–	Flight-disqualified	–
15	48	M	Never	No	Jet fighter	Absent	Right parietal lobe (cortical involvement), left frontal lobe (subcortex)	No	6 mm, 3 mm	Sporadic	Multiple lesions	No	II, III	Normal	Conservative management	High risk	High risk	Flight-disqualified	–
16	21	M	Never	No	Jet fighter	Absent	Right frontal lobe (cortical involvement)	No	7 mm	Sporadic	Single lesion	No	III	Normal	Conservative management	–	High risk	Flight-disqualified	–
17	24	M	Never	No	Jet fighter	Absent	Right frontal lobe (cortical involvement)	No	11 mm	Sporadic	Single lesion	No	II	Normal	Conservative management	High risk	High risk	Flight-disqualified	–
18	24	M	Never	No	Jet fighter	Absent	Right cerebellum	No	13 mm	Sporadic	Single lesion	Yes	II	Normal	Conservative management	High risk	–	Flight-disqualified	–
19	25	M	Never	No	Helicopter	Absent	Right temporal lobe (cortical involvement)	No	9 mm	Sporadic	Single lesion	No	II	Normal	Conservative management	High risk	High risk	Flight-disqualified	–
20	26	M	Never	No	Jet fighter	Absent	Left occipital lobe (cortical involvement)	No	5 mm	Sporadic	Single lesion	No	III	Normal	Conservative management	-	High risk	Flight-disqualified	–
21	27	M	Never	No	Transport aircraft	Absent	Pons	Brainstem	5 mm	Sporadic	Single lesion	No	III	Normal	Conservative management	High risk	–	Flight-disqualified	–
22	33	M	Never	No	Jet fighter	Absent	Pons	Brainstem	-	Sporadic	Single lesion	No	IV	Normal	Conservative management	High risk	–	Flight-disqualified	–
23	22	M	Never	No	Jet fighter	Limb paresthesia	Spinal cord (C1-2)	–	21 mm	Sporadic	Single lesion	No	I	Normal	Surgery				
24	28	M	Never	No	Helicopter	sudden headache & dizziness	Right cerebellum	No	26 mm	Sporadic	Single lesion	No	I	Normal	Surgery				
25	44	M	Never	No	Jet fighter	Absent	Right orbital apex	No	15 mm	Sporadic	Single lesion	No							

CCM, cerebral cavernous malformation; DVA, developmental venous anomaly; EEG, electroencephalogram.

All 22 pilots were male, with a mean age of 32 years (range: 21–48), and had no history of smoking or hypertension. The cohort consisted of 19 jet fighter pilots, 2 helicopter pilots, and 1 transport aircraft pilot (see Table 2).

**Table 2:** Summary of characteristics in 22 military pilots with asymptomatic CCMs.

Variable	Value
Total number of pilots	22
Age at diagnosis (years), mean (range)	36 (21–48)
Aircraft types	
Jet flighter	19 (86.4%)
Transport aircraft	1 (4.5%)
Helicopter	2 (9.1%)
Lesion multiplicity	
Single	21 (95.5%)
Multiple	1 (4.5%)
Total number of lesions	23
Anatomical location	
Frontal lobe	12 (52.2%)
Temporal lobe	4 (17.4%)
Parietal lobe	3 (13.0%)
Occipital lobe	1 (4.3%)
Cerebellar hemisphere	1 (4.3%)
Brain stem	2 (8.7%)
Zabramski classification (*n*), mean size (range)	
Type I	0
Type II	7, 9.1 mm (6 mm−13 mm)
Type III	9, 3.9 mm (2.5 mm−7 mm)
Type IV	7, -

CCM, Cerebral Cavernous Malformation.

A total of 23 CCMs were identified among the 22 pilots (see Table 2). One pilot had multiple lesions (case 15), while the remaining had solitary lesions. All cases were sporadic CCM. The anatomical distribution of the 23 lesions was as follows: frontal lobe (*n* = 12), temporal lobe (*n* = 4), parietal lobe (*n* = 3), occipital lobe (*n* = 1), cerebellar hemisphere (*n* = 1), and brainstem (*n* = 2). Five lesions involved the cerebral cortex, one was located in the precentral gyrus, and one was associated with a developmental venous anomaly (DVA). Based on the Zabramski classification (4), the lesions were distributed as follows: 7 Type II (mean size: 9.1 mm; range: 6–13 mm), 9 Type III (mean size: 3.9 mm; range: 2.5–7 mm), and 7 Type IV (size not routinely measurable). No Type I lesions were identified.

### 3.2 Aeromedical decision-making

A multidisciplinary team composed of neurology, neurosurgery, and aerospace medicine specialists assessed the in-flight incapacitation risk for these 22 military pilots with asymptomatic CCMs. Six pilots were permanently disqualified from flight duties due to a high risk of symptomatic hemorrhage leading to in-flight incapacitation; this group included two with brainstem CCMs, two with Zabramski Type II CCMs located within the white matter of a cerebral lobe, one with a cerebellar CCM accompanied by a DVA, and one with a precentral gyrus CCM. An additional five pilots, whose CCMs involved the cerebral cortex, were also disqualified due to a high risk of epilepsy causing in-flight incapacitation. For the remaining 11 pilots, the multidisciplinary team concluded that the risk of in-flight incapacitation was negligible (approximately 0%), and thus their flying status was approved without restrictions.

Annual case-by-case inspections by specialists are mandated and must incorporate provisions for MRA surveillance and psychological support. Post-certification, 11 pilots flew safely for mean 34 months (range: 12–53 months). Follow-up MRI performed after more than 1 year demonstrated that the CCM morphology remained stable, with no evidence of growth or hemorrhage, in all 11 pilots.

## 4 Discussion

CCMs are vascular anomalies characterized by clusters of dilated, thin-walled capillaries prone to microhemorrhages (1). The predominant clinical manifestations of CCMs include seizures (50%), symptomatic intracranial hemorrhage (25%), and focal neurological deficits without recent radiographic evidence of bleeding (25%) (1, 2). Critical findings from Josephson et al.'s (6) study of 23 CCM patients presenting with seizure onset revealed a 94% probability of seizure recurrence within a 5-year period, and most of the events occur within the year following the initial seizure. Thus, almost all CCMs patients with first-ever seizure but no prior hemorrhage will develop epilepsy (7). More significantly, for cases in which hemorrhage constitutes the initial presentation, the rebleeding risk during the subsequent 1–5 years ranges from 14% to 56% (2, 8). These substantial neurological risks represent an unacceptable operational hazard for military flight personnel, rendering symptomatic CCM patients unfit for flight duties.

However, the widespread implementation of advanced neuroimaging techniques, particularly SWI in routine cranial MRI protocols, has resulted in approximately 40–50% of CCM diagnoses being incidental findings in asymptomatic individuals (9–12). The aeromedical evaluation of these asymptomatic cases presents a significant challenge, requiring comprehensive and systematic assessment of their risks for both seizure and intracranial hemorrhage.

### 4.1 CCM-related epilepsy

CCMs lack neural tissue and thus are not intrinsically epileptogenic (1, 7, 8, 11). Current evidence suggests that CCM-related epilepsy arises from recurrent microhemorrhages or a first symptomatic hemorrhage, leading to perilesional hemosiderin deposition, reactive gliosis, and chronic inflammatory changes within adjacent brain parenchyma (2, 7, 13–15). A meta-analysis by Taslimi et al. (16) reported an annual incidence rate of first-ever seizures of 1.5% among CCMs patients; however, this estimate included both symptomatic and familial cases. In contrast, a long-term prospective cohort study (mean follow-up of 12.5 years) of incidental CCMs (*n* = 107) observed no incident seizures during follow-up (17). Furthermore, a population-based prospective study demonstrated that the cumulative 5-year risk of first-ever seizure was 4% in patients with incidental CCMs and 6% in adults presenting with intracranial hemorrhage or focal neurological deficits, with no significant difference between the two groups (6). It should be noted that the under-ascertainment of incidental CCMs, owing to the omission of numerous asymptomatic individuals, means this study provides an upper estimate of the true first-ever seizure risk in this patient population (6, 18). Some studies suggest that the risk of CCM-related epilepsy exhibits a positive correlation with disease duration, increasing by approximately 1–2% per year (7). Meanwhile, a meta-analysis also showed equivalent seizure risks in familial vs. sporadic CCM cohorts (pooled incidence 1.5% per patient-year) (16).

Emerging evidence has identified several independent risk factors for seizure development, including lesion multiplicity, cortical involvement and archicortical or mesiotemporal localization, exclusively subcortical CCMs are highly unlikely to cause epilepsy (2, 13, 14, 19–21). The relationship between the size of CCMs or surrounding hemosiderin rings and epilepsy remains controversial. According to Zhang et al.'s (21) study, a hemosiderin rim encircling the CCM lesion correlates with the emergence of CCM-related epilepsy; however, this correlation was not supported by another study by Menzler et al. (19). In his study, Menzler et al. (19) suggested an increased likelihood of secondary epilepsy in patients with increased CCM lesion size, whereas Josephson's (6) research found no correlation. Meanwhile, Chou et al. (22) demonstrated that the risk of CCM-related epilepsy may be associated with both the volume and proportion of gray matter displacement caused by the lesion. Concurrently, it is also noteworthy that some studies have indicated a higher likelihood of epilepsy development when the CCM is located in the left hemisphere (19).

In our cohort, none of the 23 CCMs in the 22 military pilots were located in the archicortical or mesiotemporal regions. However, five CCMs were found to involve the cerebral cortex. Among these five CCMs, three were Zabramski type II and two were type III, with a mean size of 7.6 mm (range: 5 mm−111 mm). Based on the aforementioned clinical evidence, we reasoned that these five CCMs carried a higher risk of CCM-related epilepsy.

### 4.2 CCM-related symptomatic hemorrhage

CCMs can occur in any region of the brain, with approximately 66% located in the cerebral hemispheres, 20% in the brainstem, 6% in the cerebellum, and about 8% in the basal ganglia or deep nuclei (1). A meta-analysis by Gross et al. (23), which included 12 studies on the natural history of CCMs, reported an annual hemorrhage rate of 2.5% per patient-year (range 1.3%−5.1%) over 5081.2 patient-years of follow-up; however, most bleeding events are asymptomatic microhemorrhages. Furthermore, a long-term natural history study of CCM patients found that incidentally identified asymptomatic lesions had a significantly lower annual first hemorrhage rate of only 0.08% per patient-year (17). Meanwhile, Smith et al. noted that the annual bleeding risk for sporadic, incidentally discovered CCMs ranges between 0.1% and 1.0% (1). Whereas familial CCM cases exhibit a higher annual hemorrhage rate of approximately 4%, with about 60% of patients experiencing symptomatic bleeding and 32%−60% developing seizures (1, 2).

The risk of intracranial symptomatic hemorrhage in CCM patients is primarily influenced by prior hemorrhageand and re-hemorrhage is considerably more common during the first 2 years after the first bleeding (2, 11, 23–26). Kondziolka et al. (27) reported in their prospective observational study that the annual hemorrhage rates were 4.5% and 0.6% in patients with and without previous hemorrhage history, respectively.

Brainstem location of the CCM is another important risk factor for symptomatic hemorrhage (28). A meta-analysis by Horne et al. (29), which included 22 studies, demonstrated that brainstem CCMs carry a 5-year hemorrhage risk of 8%, significantly higher than the 3.8% risk observed in non-brainstem lesions. Similarly, Taslimi et al. (30) conducted another meta-analysis of 25 studies and found that non-brainstem CCMs had an annual hemorrhage risk of 0.3%, compared to 2.8% for brainstem CCMs. It is important to note that even minor hemorrhages in brainstem cavernous malformations can lead to severe and often irreversible neurological deficits (31). Furthermore, a multicenter retrospective study has demonstrated that infratentorial CCMs exhibit significantly higher hemorrhage rates compared to supratentorial lesions (32, 33). Patients with infratentorial CCMs carry a 3 to 4-fold increased risk of symptomatic hemorrhage relative to those with supratentorial CCMs, indicating that infratentorial lesions may follow a more aggressive clinical course (33).

Meanwhile, several retrospective studies have demonstrated that large CCMs or brainstem lesions with a diameter ≥10 mm are both associated with a significantly increased risk of hemorrhagic events (32, 34, 35). In contrast, the hemorrhage rate of dot-like cavernomas was 0.7% per lesion-year (36). Moreover, a recent retrospective multicenter case-control study by Scerrati et al. (37) also identified that a lesion volume ≥300 mm3 was a significant predictor of hemorrhage in CCMs. However, some studies argue that CCM size does not correlate with hemorrhage risk (23, 38).

Moreover, CCMs accompanied by DVA has been associated with an increased hemorrhagic risk in clinical studies, a phenomenon potentially attributable to impaired venous drainage and consequent hemodynamic stress (39–41). Meanwhile, some observational retrospective studies have demonstrated that CCMs with associated DVA are not associated with an increased risk of intracerebral hemorrhage. During untreated 5-year follow-up periods, the hemorrhage risk in these patients was comparable to that in CCM patients without DVA (23, 42). Concurrently, prospective studies indicate symptomatic hemorrhage risks vary significantly by Zabramski classification: Type I CCMs demonstrate the highest hemorrhage rate (13.9%), followed by Type II (2.9%) and Type III (1.8%) (43). Type IV lesions show no symptomatic hemorrhage events, reinforcing their characterization as incidental findings with benign clinical behavior (36, 43–45).

It is noteworthy that although CCMs typically bleed in a low-pressure and localized manner within the lesion or adjacent brain tissue, the risk of symptomatic hemorrhage is significantly higher if the lesion is located in an eloquent area compared to non-eloquent areas (46).

In summary, the following lesions in our cohort collectively presented a higher risk of symptomatic hemorrhage: two brainstem CCMs, one precentral gyrus CCM, one cerebellar CCM with an associated DVA, and seven Zabramski Type II CCMs.

### 4.3 Aviation medical certification

The impact of routine aviation stressors, including acceleration forces (G-forces), hypobaric conditions, vibration, noise, and electromagnetic radiation, on the natural progression of cerebral CCMs in aviators remains unclear. In particular, it remains unknown whether the high-stress training environment of flight, where positive acceleration (+Gz) exposure reduces cerebral arterial pressure while the Anti-G Straining Maneuver (AGSM) transiently elevates intra-thoracic and intra-abdominal pressure, increases the risk of CCM hemorrhage (47, 48). Thus, theoretically, the unique occupational exposure profile of military pilots may elevate their risk of CCM-related epilepsy and symptomatic hemorrhage compared to the general population. Consequently, the aeromedical evaluation of CCMs remains a significant challenge in aviation medicine, and there is currently a lack of standardized protocols for granting medical clearance to military pilots with asymptomatic CCMs (3, 5).

Aeromedical decision-making for military pilots currently lacks a clear, evidence-based framework due to these unresolved issues. A widely referenced safety standard is the ‘1% rule,' which posits an annual acceptable risk of medical incapacitation at 1% (equivalent to 1 in 100 person-years), a threshold designed to ensure no more than one fatal accident per 107 flight hours (49). Notably, this guideline was specifically established for commercial operations with dual-pilot crews. However, military pilots face more stringent medical certification standards than civilian aviators due to the high-intensity, complex, and risky nature of military flight operations.

Given that an epileptic seizure can lead to complete in-flight incapacitation, our risk assessment for this outcome is exceptionally stringent. Among the five cases with CCMs involving the cerebral cortex, two were Zabramski type III lesions. Although these two lesions were smaller than 10 mm and showed no evidence of hemosiderin deposition or perilesional edema, we nonetheless considered them to pose a high risk of in-flight incapacitation. Consequently, the affected pilots were permanently disqualified from flight duties.

Meanwhile, in contrast to the typically more severe neurological deficits caused by arterial pathologies such as arteriovenous malformations or aneurysms, hemorrhages associated with CCMs are generally minor, predominantly presenting as micro-hemorrhages. Most are asymptomatic or manifest only as headache attributed to the hemorrhage. In our cohort, after excluding CCMs with high risks of symptomatic hemorrhage or epilepsy, 11 lesions remained (five Zabramski Type III and six Type IV). We deemed the risk of in-flight incapacitation from these lesions to be nearly 0%, and consequently, all 11 pilots were approved for unrestricted flight status, despite existing literature reports of hemorrhage in Type IV lesions (50). Post-certification, 11 pilots flew safely for mean 34 months.

During this evaluation process, we established the following certification criteria: First, symptomatic CCMs warrant disqualification from flight duties. Second, for lesions located in the archicortical or mesiotemporal regions, or those involving the cerebral cortex, the risk of seizure-induced in-flight incapacitation is considered high, necessitating grounding. Third, lesions situated in eloquent areas (including the brainstem), those that are multiple, those associated with a DVA, and Zabramski type I and II CCMs are assessed as having a high risk of in-flight incapacitation due to symptomatic hemorrhage and also require permanent disqualification. Four, solitary Zabramski type III or IV CCMs that do not involve the cortex, are not associated with a DVA, and are located in non-eloquent areas, are judged to carry a nearly negligible risk of CCM-related complications. Consequently, aviators with such lesions may be considered for unrestricted flying status. The newly implemented protocol for individualized aeromedical waivers has allowed 11 military pilots with CCMs—six of whom are highly experienced with >1,500 flight hours—to return to flying status. This marks a departure from China's previous policy of mandatory grounding for all pilots upon CCM diagnosis, regardless of symptoms, which would have disqualified every pilot in this cohort (3).

Additionally, pilots with CCMs require periodic reassessment with brain MRI and EEG monitoring, given that a body of research demonstrates the dynamic nature of CCMs—including size changes and *de novo* lesion formation—and their association with increased hemorrhage risk and cumulative neurological deficits. A prospective volumetric analysis of 107 CCMs revealed that during a mean follow-up of 3.7 years, 43% of lesions increased in size, 35% decreased, and 22% remained stable (51). Moreover, a retrospective study found that changes in CCM size during follow-up were associated with higher rates of hemorrhagic events (32, 52, 53). Another study indicated approximately 4% of sporadic CCM patients develop *de novo* lesions within a 2-year period (51). Meanwhile, repeated hemorrhages from CCMs can cause varying degrees of neurological impairment that follow a cumulative pattern over time (23). A recent study by Wadhwa et al. (54) indicated that for patients managed conservatively, the most cost-effective MRI follow-up strategy for brainstem CCMs is annually, while for non-brainstem CCMs, follow-up every 3 years is generally the most economical. For military aircrew, who are required to undergo annual routine physical examinations, it is reasonable to incorporate annual brain MRI (including SWI) and electroencephalogram (EEG) monitoring into their follow-up protocol.

Meanwhile, several studies indicate that patients with untreated CCMs may exhibit concerns regarding symptomatic hemorrhage or *de novo* CCM development, consequently demonstrating significantly higher anxiety levels compared to the general population. This further underscores the critical need for mental health guidance for military aviators with untreated CCMs during flight operations (55–58). Given the well-documented association between a diagnosis of CCM and clinically significant anxiety, providing systematic mental health support is essential for the care of military aviators with this condition. Consequently, our institution has adopted a formal psychological support protocol. This framework incorporates routine screening via the Hospital Anxiety and Depression Scale (HADS) as part of the initial aeromedical assessment. Any aviator obtaining a HADS score of 8 or higher, or who expresses considerable distress, is automatically scheduled for evaluation and counseling with a specialized aviation psychologist. To guarantee ongoing adherence, this referral mechanism is integrated into the mandatory yearly medical examination process.

The protocol is grounded in systematic risk analysis conducted by our center, focusing on epilepsy, hemorrhage, and in-flight incapacitation. This proactive, evidence-based framework aims to minimize operational impact and may serve as a global reference.

### 4.4 Limitation

This investigation contributes one of the largest single-center cohorts in the literature on aeromedical certification for military aviators with asymptomatic CCMs, by providing substantial practical evidence for waiver guidance. And, several limitations in this study should be acknowledged. First, the duration of observation in our study is insufficient to permit a conclusive evaluation of the long-term accuracy of aeromedical clearances granted to individuals with asymptomatic CCMs. Second, although quantitative susceptibility mapping (QSM) has recently demonstrated promising utility in stratifying the risk of epilepsy and hemorrhage in CCMs (59–61), this technique was not employed in our center. Consequently, the absence of QSM data may have limited the objectivity and accuracy of our risk assessment. Third, the cohort included seven cases of Zabramski Type IV CCMs. On SWI, these lesions can be difficult to distinguish from microhemorrhages or capillary telangiectasias, creating a potential for misdiagnosis that could impact the aeromedical disposition.

## 5 Conclusion

Our experience demonstrates that military pilots with CCMs may be granted conditional medical clearance following prudent evaluation by a multidisciplinary expert panel. During this process, a preliminary certification framework has been established. This framework will require future validation and refinement, with continued monitoring and outcome reporting for those approved to return to flight duties. As the largest reported cohort study of aviators with CCMs, we hope these findings may provide a reference for aeromedical decision-making in military aviation globally.

## Data Availability

The original contributions presented in the study are included in the article/supplementary material, further inquiries can be directed to the corresponding authors.
